# Synchronous Parotid and Homolateral Submandibular Gland Pleomorphic Adenoma

**Published:** 2019-05

**Authors:** Anup Singh, Aru-Chhabra Handa, Ritesh Sachdev

**Affiliations:** 1 *Department of Otolaryngology and Head and Neck Surgery, Medanta- The Medicity, Gurugram, India.*; 2 *Department of Pathology, Medanta- The Medicity, Gurugram, India* *.*

**Keywords:** Pleomorphic adenoma, Salivary glands, Synchronous, Surgical treatment

## Abstract

**Introduction:**

Pleomorphic adenomas are benign neoplasms of salivary glands. The simultaneous homolateral occurrence of these tumors in salivary glands is exceedingly rare.

**Case Report:**

An adult female presenting to our OPD with the swelling of right-sided preauricular and submandibular regions was diagnosed with the pleomorphic adenoma based on fine needle aspiration cytology. The patient was subjected to the excision of both swellings under general anesthesia. Postoperative facial nerve functions were within normal limits and final histopathology confirmed pleomorphic adenoma involving both the sites. A pertinent detailed literature review of English and non-English studies was indicative of only nine such cases.

**Conclusion:**

Simultaneously occurring pleomorphic adenoma involving homolateral parotid and submandibular glands is a rare phenomenon that should be kept in mind when examining the swelling of the unifocal salivary gland.

## Introduction

Pleomorphic adenomas are the most common major salivary gland neoplasms accounting for up to 60% of salivary tumors in large series. Around 80% of these tumors arise from parotid gland, while 10% arise in submandibular gland and the remaining ones arise from sublingual and minor salivary glands spread over the entire upper aerodigestive tract (1). 

Multiple primary pleomorphic adenomas are rare lesions, which mostly affect the parotid gland, either as multifocal unilateral or unifocal bilateral tumors (2,3). Simultaneously occurring pleomorphic adenomas involving both parotid and submandibular glands are exceedingly rare and nine cases have been reported in the literature till date. 

Herein we presented a case of synchronous pleomorphic adenoma involving the parotid and ipsilateral submandibular glands. Our findings highlighted the importance of exercising caution during the examination of a single salivary gland swelling and gaining awareness regarding this uncommon possibility. Moreover, a pertinent comprehensive literature review was presented.

## Case Report

A 35-year-old woman presented to the ear, nose, and throat outpatient department of the hospital with the swellings of right-sided neck, one in the region of the angle of the right jaw and the other below the right jaw (corresponding to submandibular region). She first noticed these swellings about 2-3 years ago when they were about the size of a bean. The swellings gradually progressed in size. There was no history of pain, fever, trauma, difficulty in mouth opening or facial nerve weakness. There were no comorbidities or history of addictions. On examination, she was found to have swellings present in relation to the right parotid and submandibular regions. The parotid swelling was about 2.5×2.5 cm in size, non-tender, firm, lobulated, mobile with normal overlying skin.

**Fig 1:**
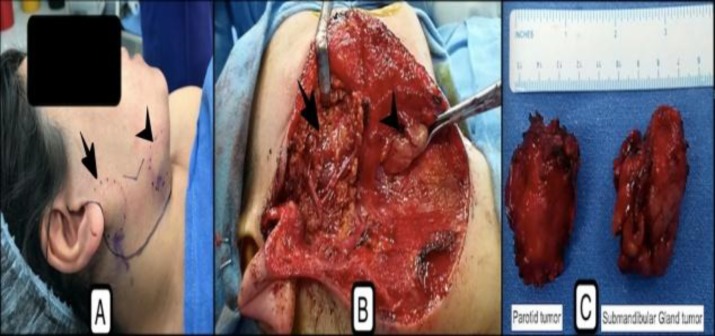
(A) Well-defined, slightly lobulated, firm mass lesion involving right parotid (arrow) and submandibular gland region (arrowhead) (encircled by dotted blue lines), (B) Intraoperative view showing the parotid bed, post superficial parotidectomy (arrow) with intact facial nerve, and submandibular gland tumour (arrowhead) in the process of removal, (C) Post-excision specimen indicating both the tumors excised in entirety with a surrounding cuff of normal tissue

 The swelling in the submandibular area was also about 2×2 cm in size, non-tender, firm, lobulated, mobile with normal overlying skin (Figure 1a). Facial nerve functions were within normal limits. Mouth opening was normal and there was no bulge in tonsillar fossa or the floor of the mouth.

Fine needle aspiration cytology (FNAC) from both the lesions showed a cellular smear with abundant chondromyxoid matrix associated with singly scattered and poorly cohesive myoepithelial cells with plasmacytoid appearance. There was no atypia. The features were suggestive of a pleomorphic adenoma. 

**Fig 2:**
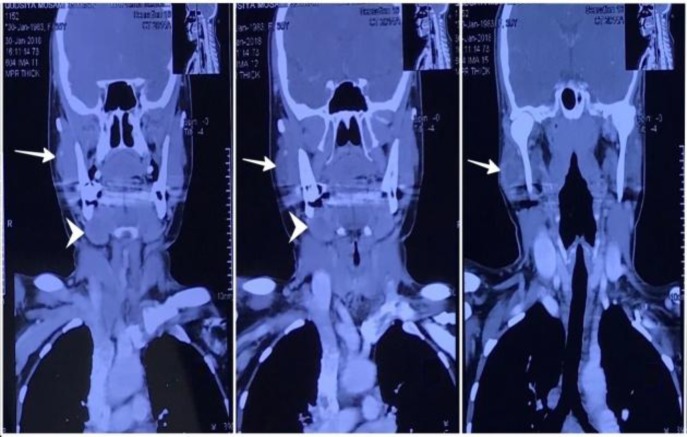
A 3.2×2.5-cm well-defined, slightly lobulated, iso to hypodense lesion with small areas of calcific foci involving the right superficial lobe of the parotid gland (white arrow) with mild focal heterogeneous contrast enhancement and central hypodense areas, and another 2.5×2.1 cm well defined, smoothly marginated iso to hypodense, poorly enhancing oval lesion involving right submandibular gland (arrowhead), causing smooth expansion of the gland (Tumor appearance is suggestive of benign etiology involving both the parotid and submandibular glands

A contrast-enhanced computed tomography (CT) scan of face and neck showed a well-defined, slightly lobulated mass lesion involving right parotid gland, suggestive of mild heterogeneous contrast enhancement. Another well-defined smooth marginated tumor with no significant contrast enhancement involved right submandibular gland. The radiographic appearance of tumors in both the locations was suggestive of a benign pathology (Fig.2).

With the presumptive diagnosis of pleomorphic adenoma of right parotid and submandibular region, the patient was planned for right-sided superficial parotidectomy and right submandibular gland excision under general anesthesia. A modified Blair incision with cervical extension was performed to surround the submandibular area. The superficial parotidectomy and submandibular gland excision were performed after raising skin and subplatysmal flaps, which preserved the function of the facial nerve and its branches (Fig.1b). Intraoperatively, the tumors in both the locations were firm and lobulated. The treatment policy was the excision of entire tumors, superficial lobe of the parotid gland, and entire submandibular gland (Figure 1c). Postoperative period was uneventful and facial nerve functions were normal. 

**Fig 3:**
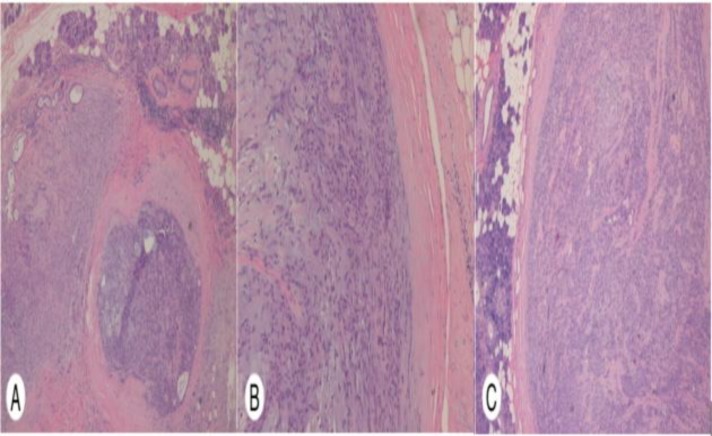
(A) Parotid gland: Two encapsulated biphasic tumor nodules with normal salivary gland tissue at the periphery. (H & E; 10×), (B) Parotid gland: Section from tumour showing the epithelial-myoepithelial component surrounded by chondromyxoid stroma. (H & E; 20×), (C) Submandibular gland: Section indicating encapsulated biphasic tumor surrounded by normal tissue, comprising predominantly of the epithelial-myoepithelial component. (H & E; 10×).

On gross pathological examination, the parotid and submandibular gland tumors were measured 34×25×22 mm and 45×25×20 mm, respectively. Serial slicing of the tumor showed grey white firm well-circumscribed lesion with pushing margins, with a surrounding rim of normal salivary gland tissue. Microscopic inspection showed both the tumors were surrounded by a capsule. The biphasic population of epithelium and mesenchymal cells were observed with epithelial cells in glands and myoepithelial cells embedded in the chondromyxoid stroma. The investigated regions had no necrosis or significant atypia. The final histopathological diagnosis of parotid and submandibular gland pleomorphic adenoma was performed postoperatively (Fig.3).

## Discussion

The multifocal salivary gland tumors are uncommon lesions, which mostly affect the parotid gland. The most common type of these tumors are Warthin’s tumors followed by pleomorphic adenomas (4). 

Synchronous pleomorphic adenomas involving both parotid and submandibular glands are rare, and till date, only 9 such cases have been reported including both English and non-English (German and Japanese) studies (5-13). 

Synchronous tumors are the ones which are presented and identifies simultaneously in one patient, while the metachronous tumors refer to tumors detected to be present in different sequential time frames. 

Metastatic pleomorphic adenomas are regarded as harboring low-grade malignant potential, and a total of 83 such cases have been documented in the literature so far (14).

 In most cases, the metastatic tumors are found in the setting of recurrent tumors at the original wound bed, and have been hypothesized to be a result of tumor spill resulting in lymphovascular seeding at the time of primary excision. In our case, the synchronous tumors appear to be two independent primary tumor foci with no history of surgery or trauma leading to seedling. 

**Literature review**

A rigorous literature search was conducted using Pubmed, Embase, and Google Scholar databases. The embedded references were analyzed for any additional reports. The details of the patients are mentioned in Table 1.

**Table 1:** Literature Review

**Case no. (ref)**	**Year of the report of the case**	**Age (year)/ Gender**	**Side**	**Presentation**	**Imaging**	**Treatment**	**Outcome and F/U**
1. (ref.5)	1956	62/F	Rt Parotid PA Rt SMG PA	3×3 cm tumor in a superficial lobe of parotid x 11 years 4×6.5 cm tumor involving Rt SMG×11 years	NA	Rt SP Rt SMG excision	NA
2. (ref.6)	1966	58/F	Rt Parotid PA Lt SMG PA	3×3×4 cm tumor in a superficial lobe of parotid×18 months 2×2×3 cm tumor involving SMG×10 years	Soft tissue roentgenogram of face and neck	Rt SP Lt SMG excision	No recurrence after 21 months follow up
3. (ref.7)	1987	68/F	Rt SMG PA Rt Parotid PA	6.5×3×3 cm x 4 years4×3.5×2 cm dumbbell shaped deep lobe tumor ×10 years	CECT with sialography Tc 99^m^ scintigraphy of salivary glands	Rt SMG tumor excision Rt Total Parotidectomy	NA
4. (ref.8)	1999	52/F h/o radiation exposure in childhood	Rt SMG PA Rt Parotid PA	Both tumors 2×2 cm in size, present for 15-20 years	CECT FNAC	Concomitant Rt SP and SMG excision	NA
5. (ref.9)	2001 (Japanese)	29/M	Rt Parotid PA Lt SMG PA (discovered on imaging)	34×25 mm for 3 years 10 mm diameter	CEMRI FNAC	Concomitant Rt SP and Lt SMG excision	NA
6. (ref.10)	2005 (German)	59/?	Rt Parotid PA Rt SMG PA	3.4 cm for 5 years 2.8 cm for 1 year	CEMRI FNAC	Rt SP and Rt SMG excision	Complete excision, no recurrence at 2 years F/U
7. (ref.11)	2010	57/F	Lt Parotid PA Lt SMG PA	One year duration	?	Lt SP and Lt SMG excision	?
8. (ref.12)	2015	22/F	Rt (parotid + SMG) Lt (parotid)	Right side parotid PA – 2×2 cm for 1 year in 2005 Right SMG PA 2×2 cm in 2007 Lt Parotid PA 2×2 cm in 2012	CECT for 2^nd^ and 3^rd^ tumor FNAC for all the three tumors	Rt SP Rt SMG excision Lt SP	Complete excision. No recurrence at the operated site until 1 year after last procedure
9. (ref.13)	2017 Preceded by URI	30/M	Rt (SMG) Lt (parotid)	Rt SMG PA – 26 mm Lt Parotid PA – 22 mm The two tumors diagnosed 5 months apart	USG CECT FNAC	Concomitant Rt SMG excision and Lt SP	Complete excision, F/U NA

(Rt- right, Lt- left, M- male, F- female, PA- pleomorphic adenoma, SMG-submandibular gland, NA- not available, SP-superficial parotidectomy, F/U- follow up, URI- upper respiratory tract infection, CECT–contrast-enhanced CT, CEMRI–contrast-enhanced magnetic resonance imaging, FNAC-fine needle aspiration cytology)

Patients were within the age range of 22-69 years. This lesion affect women more than men (3:1), while gender information could not be obtained in one case. Five patients had the involvement of homolateral salivary gland and three had the involvement of Parotid and contralateral SMG. One patient had a triple tumor involving bilateral parotid and right side submandibular gland metachronously. The size of the parotid gland ranged 2-4 cm while that of the SMG ranged from 1 (discovered accidentally on imaging) to 6.5 cm. Most of the cases were of long standing duration (the longest duration of the tumor was within the range of 15-20 years before presentation). The facial nerve was reported to be normal in all the reported cases. As a part of the preoperative evaluation, FNAC was performed in five cases preoperatively, which indicated benign mixed tumors. Contrast-enhanced CT was performed for four cases, which was the most commonly used imaging investigation with additional sialography in one case. In one of the patients, the SMG tumor was discovered in the Contrast CT conducted for opposite parotid PA. Contrast-enhanced magnetic resonance imaging was conducted for two cases, while ultrasonography and X-ray soft tissue face and neck (to look for calculus) were performed each for one case. One of the patients received technetium-99m scintigraphy. All the patients were subjected to standard surgical procedures in the form of Superficial Parotidectomy and SMG excision via a transcervical approach, while one patient underwent total conservative parotidectomy for tumor involving deep lobe of the parotid gland. Follow-up sessions were available for only three patients with no recurrence after a 2-year follow up. 

In addition to these well-documented cases, Turnbull and Frazell reported one case of bilateral benign mixed (parotid and contralateral submaxillary) tumor in their series of 27 patients with different tumors of major salivary glands (15). However, no further details of the patient were mentioned.

Frazell, Foote, and Lenson mentioned a patient of Slaughter with parotid pleomorphic adenoma and homolateral submaxillary pleomorphic adenoma as personal communications; however, no further details of the patient could be obtained (16,17,5). Another case of multiple mixed tumors involving both parotid and submandibular gland was reported by Sakima (1938) in Germany as cited by Yajin et al. (7). However, the abstract or full text of this study could not be retrieved during the literature search.

McGrath reported concomitant right parotid and SMG tumor in a 56-year-old male patient with intact facial nerve function (18), FNAC, and frozen sections showing a benign mixed tumor. The final histopathology showed a premalignant disease in parotid tumor and early pre-invasive changes in SMG tumor. 

Radiation was introduced as a risk factor for pleomorphic adenomas, which may result in multiple primaries in the exposed salivary gland tissues as a field tumor predisposition. Nagler et al. considered radiation as a possible predisposing factor for the occurrence of simultaneous major salivary gland tumors (8). In our case, no such history could be obtained.

Cytogenetic analysis of pleomorphic adenomas revealed that up to 70% of these tumors were associated with some genetic mutation, commonly involving the locus 8q12 and 12q13-15 (19). Although it would be of interest to study the role of genetic influences in multiple PA, no study has paid attention to this issue.Shinohara et al. mentioned that heterotopic salivary gland tissue located in the lymph nodes in the parotid and submandibular region can also cause multifocal tumor (20). The tumors in these cases were lined by a thin periphery of lymphoid tissue in entire circumference. In our case, the tumor had well-defined capsulated surface without surrounding lymph node tissue.

Complete excision of the tumor with an adequate cuff of surrounding normal tissue remains the treatment of choice. This, in case of parotid tumors, is achieved by a superficial parotidectomy or total conservative parotidectomy depending on the location and extent of the tumor. However, the removal of whole gland along with the tumor is suggested in the cases of submandibular gland tumors, which ensures complete disease removal. Regarding complete excision, there appears to be no higher risk of recurrence in these tumors when compared to unifocal tumors.

## Conclusion

The current case and the literature review emphasized the need for thorough head and neck examination, including the other salivary glands in the common case scenario of unifocal pleomorphic adenomas to rule out a synchronous tumor. After complete excision, vigilance on the part of clinician and patient need to be exercised during follow-up. Moreover, it should be kept in mind that there is a possibility of a metachronous tumor in other major or minor salivary glands. 
